# Enhancing Clinical History Taking Through the Implementation of a Streamlined Electronic Questionnaire System at a Pediatric Headache Clinic: Development and Evaluation Study

**DOI:** 10.2196/54415

**Published:** 2024-11-08

**Authors:** Jaeso Cho, Ji Yeon Han, Anna Cho, Sooyoung Yoo, Ho-Young Lee, Hunmin Kim

**Affiliations:** 1 Department of Pediatrics, Seoul National University Bundang Hospital, 166 Gumi-ro, Bundang-gu, Seongnam, 03080, Republic of Korea, 82 317877297; 2 Department of Pediatrics, Inha University Hospital, Incheon, Republic of Korea; 3 Healthcare ICT Research Center, Seoul National University Bundang Hospital, Seongnam, Republic of Korea; 4 Department of Nuclear Medicine, Seoul National University College of Medicine, Seoul, Republic of Korea; 5 Department of Pediatrics, Seoul National University College of Medicine, Seoul, Republic of Korea

**Keywords:** electronic questionnaire system, electronic questionnaire, history taking, medical history, headache, migraine, neuralgia, pediatric, paediatric, infant, neonatal, toddler, child, youth, adolescent

## Abstract

**Background:**

Accurate history taking is essential for diagnosis, treatment, and patient care, yet miscommunications and time constraints often lead to incomplete information. Consequently, there has been a pressing need to establish a system whereby the questionnaire is duly completed before the medical appointment, entered into the electronic health record (EHR), and stored in a structured format within a database.

**Objective:**

This study aimed to develop and evaluate a streamlined electronic questionnaire system, BEST-Survey (Bundang Hospital Electronic System for Total Care-Survey), integrated with the EHR, to enhance history taking and data management for patients with pediatric headaches.

**Methods:**

An electronic questionnaire system was developed at Seoul National University Bundang Hospital, allowing patients to complete previsit questionnaires on a tablet PC. The information is automatically integrated into the EHR and stored in a structured database for further analysis. A retrospective analysis compared clinical information acquired from patients aged <18 years visiting the pediatric neurology outpatient clinic for headaches, before and after implementing the BEST-Survey system. The study included 365 patients before and 452 patients after system implementation. Answer rates and positive rates of key headache characteristics were compared between the 2 groups to evaluate the system’s clinical utility.

**Results:**

Implementation of the BEST-Survey system significantly increased the mean data acquisition rate from 54.6% to 99.3% (*P<*.001). Essential clinical features such as onset, location, duration, severity, nature, and frequency were obtained in over 98.7% (>446/452) of patients after implementation, compared to from 53.7% (196/365) to 85.2% (311/365) before. The electronic system facilitated comprehensive data collection, enabling detailed analysis of headache characteristics in the patient population. Most patients (280/452, 61.9%) reported headache onset less than 1 year prior, with the temporal region being the most common pain location (261/703, 37.1%). Over half (232/452, 51.3%) experienced headaches lasting less than 2 hours, with nausea and vomiting as the most commonly associated symptoms (231/1036, 22.3%).

**Conclusions:**

The BEST-Survey system markedly improved the completeness and accuracy of essential history items for patients with pediatric headaches. The system also streamlined data extraction and analysis for clinical and research purposes. While the electronic questionnaire cannot replace physician-led history taking, it serves as a valuable adjunctive tool to enhance patient care.

## Introduction

Headache is one of the most common neurological symptoms in children, with a reported prevalence of 54.4% to 58.4% in previous population-based studies [1-4]. Using an appropriate approach in the differential diagnosis of pediatric headache is critical because it can potentially impact children’s quality of life in a significant manner [5,6]. A thorough history taking is required to differentiate the symptoms of different primary headaches and to rule out a small but critical number of life-threatening secondary headaches, such as brain tumors or intracranial hypertensions [7].

Despite its importance, many studies have shown that history taking can be the major contributing factor in the misdiagnosis of pediatric headache due to miscommunications and limited time available, leading to missing key information in a real-world setting [8,9]. To overcome such obstacles, various questionnaires for efficient history taking and early detection of specific types of primary headaches have been developed in the past, such as the Diagnostic Headache Diary [10], ID Migraine [11], Migraine Screen Questionnaire [12], and Brief Headache Screen [13]. Even with the development of different history-taking tools, a paper-based, self-administered questionnaire itself poses a significant limitation in data collection and organization since it requires laborious, time-intensive, manual input [14]. Consequently, there has been a pressing need to establish a system whereby the questionnaire is duly completed before the medical appointment, entered into the electronic health record (EHR), and stored in a structured format within a database.

Numerous studies across multiple clinical fields comparing the effectiveness of paper-based and computer-based questionnaires have consistently shown that electronic health history questionnaires have higher usability scores and are more cost-effective than paper-based ones [14-20]. In this study, we developed an electronic questionnaire system named BEST-Survey (Bundang Hospital Electronic System for Total Care-Survey) that enables a streamlined previsit electronic questionnaire, with automatic integration into the EHR to aid clinicians’ history taking and the construction of a structured database for further data analysis. This study aimed to develop and evaluate the BEST-Survey system among patients with pediatric headaches.

## Methods

### Electronic Questionnaire System (BEST-Survey) Development

At Seoul National University Bundang Hospital, we constructed an electronic questionnaire system named BEST-Survey to enhance the clinical utility of patient-provided medical information. Using the BEST-Survey system, patients complete the questionnaire on a tablet PC before their visit, and the information is automatically integrated into our EHR to aid clinicians during their history taking. The patient-provided information is stored in a structured database for further data analysis.

The BEST-Survey system is composed of 2 parts: questionnaire archives and electronic questionnaire system structure. A task force comprising 30 members, including doctors, nurses, and medical information technologists, evaluated the questionnaires based on 5 criteria: predicted demand, target age group, questionnaire availability, clinical utilization within the EHR, and copyright issues. A total of 59 questionnaires were created based on the needs of 12 departments, one of which was the pediatric headache questionnaire. The electronic questionnaire system structure within BEST-Survey consists of the following components: (1) an electronic questionnaire input system, delivered using technology such as a tablet PC or computer, for patients to complete during the preclinic visit; (2) automatic integration of patient response to the EHR to aid clinicians’ history taking; and (3) construction of a structured database based on patient input for further data analysis.

### Pediatric Headache Questionnaire Development

At our pediatric neurology outpatient clinic, located in Gyeonggi Province, the largest province in South Korea by population, we see 200‐250 new patients with pediatric headaches annually. As a tertiary referral center, we primarily see patients with red-flag symptoms or refractory headaches referred from primary and secondary clinics. To improve the history-taking process and enhance clinical assessments for these patients, we developed a previsit questionnaire specifically for patients with headache attending their initial consultation. The questionnaire was developed with reference to Swaiman’s *Pediatric Neurology 5th edition* [21]. To ensure patient comprehension without guidance from medical staff, we used simple language that is easily comprehensible and provided detailed explanations for complex concepts.

The initial visit questionnaire asks about detailed characteristics of the patient’s headaches, utilizing multiple-choice questions with an option for free-text entry. To evaluate detailed headache patterns, patients were asked to provide information about their headache patterns, including location, nature, duration, frequency, recent exacerbation and its nature, frequent headache timing, and aggravating or alleviating factors. The accompanying aura and its types were also inquired to provide detailed information for migraine classification. Red-flag symptoms; family history of headache; limitation in daily activity; and previous headache diagnosis, evaluations, and treatments were also included in the questionnaire to aid clinicians in the detailed evaluation of patient’s headache history and characteristics. The questionnaire ends with an open-ended question for patients to freely write down any additional concerns or questions for the initial visit. Headache severity is rated on the Numeric Pain Rating (NRS) scale or as mild, moderate, or severe for infants, as observed by their parents. A total of 35 previsit questions were developed for integration into the BEST-Survey system. The complete questionnaire created in Korean was translated into English. Both versions are shown in Multimedia Appendix 1.

### Clinical Utilization of BEST-Survey in the Pediatric Headache Clinic

**Figure 1.**
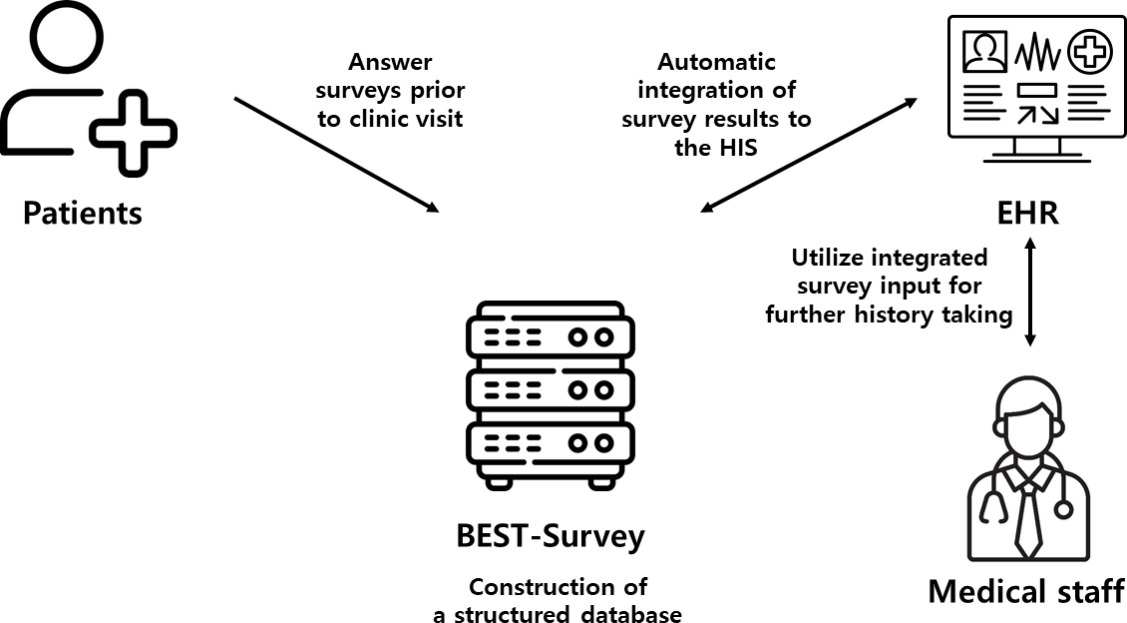
Overview of the BEST-Survey system. BEST-Survey: Bundang Hospital Electronic System for Total Care-Survey; EHR: electronic health record; HIS: hospital information system.

When the patient visited the pediatric headache clinic, trained medical staff introduced the BEST-Survey system in the waiting room and provided education on completing the headache questionnaire using a tablet PC. Patients were asked to fill out the survey with the help of their parents when necessary. The completed questionnaire was automatically integrated into the EHR, in both a free format and a structured format for the clinicians to review. The completed questionnaire was further stored into a structured database for further data analysis and tracking of the patient’s headache history. Neither the time it took for the patient to complete the survey nor the number of patients who refused to participate in the survey was recorded. Upon request for data collection, data retrieval was conducted automatically in a common data warehouse format, ensuring the exclusion of personal identifiers. Clinical information, including headache characteristics, age, sex, and other patient details, was retrieved in accordance with the preauthorized institutional review board (IRB) approval. An overview of the BEST-Survey system utilized in the pediatric headache clinic is summarized in Figure 1.

### Comparison of Acquired Clinical Information Before and After the Implementation of BEST-Survey

We conducted a retrospective analysis on the health records of patients with headaches who visited the pediatric neurology outpatient clinic at Seoul National University Bundang Hospital. The analysis included patients who visited from March 2013 to March 2015—before the implementation of the BEST-Survey system (“Before e-system group”)—and patients who visited from September 2015 to September 2017—after the implementation of the system (“After e-system group”). All new patients with pediatric headaches aged <18 years were recruited in the study, and patients were given the option to not to use the electronic questionnaire system. There was no lower age limit to the study. Patients and families who refused to use electronic questionnaire system were not included in the study. We collected information on 18 headache characteristics and associated symptoms, such as severity, onset, frequency, duration, location, characteristics, nausea, vomiting, dizziness, photophobia, phonophobia, visual aura, timing, sleep breakage, relieving factor, aggravating factor, aggravation by increased intracranial pressure, and family history in both groups. We then compared the answer rates and positive rates of 10 specific findings (onset, frequency, location, nature, duration, severity, frequent timing of the headache, factors of aggravation, associated symptoms, and visual aura) between the 2 groups to evaluate the clinical utility of BEST-Survey. The overall headache characteristics, including 10 previously mentioned, were further described to provide clinical overview our pediatric headache population group. Patients and parents were asked to complete the entire survey but were permitted to skip questions if necessary. Data analysis was processed using SPSS Statistics for Windows (version 27.0; IBM Corp). Descriptive statistics were used to express data as means, ranges, and percentages. The Mann-Whitney *U* test was used for nonparametric comparisons between the 2 groups. Statistical significance was set at *P*<.05.

### Ethical Considerations

This study was approved by the IRB of Seoul National University Bundang Hospital (B-1805-468-106). All participants were deidentified by assigning them random codes instead of hospital numbers and personal identifiers. No compensation was provided to the participants. The waiver of informed consent was approved by the IRB, as the study utilized deidentified medical records.

## Results

### Comparison of Information Achieved Before and After the Implementation of BEST-Survey

This study included 365 patients in the before e-system group and 452 patients in the after e-system group. There were no differences in onset age and sex between the 2 groups: the mean age at the visit was 9.7 (95% CI 9.4-10.2) years in the before e-system group and 10.0 (95% CI 9.7-10.4) years in the after e-system group, and 52.9% (193/365) and 49.3% (223/452) of the patients in the before and after e-system groups were female, respectively. The clinical characteristics of headaches obtained from the 2 groups were further analyzed. The mean rate of data acquisition increased significantly (*P*<.001) from 54.6% (range 27/365, 7.4% to 311/365, 85.2%) to 99.3% (range 410/452, 90.7% to 452/452, 100%) after the implementation of the e-system. The data acquisition rate for the 6 cardinal clinical features of headaches (onset, location, duration, severity, nature, and frequency) increased from a range of 53.7% (196/365) to 85.2% (311/365) in the before e-system group to a range of 98.7% (446/452) to 100% (452/452) in the after e-system group. The 3 least obtained clinical features in the before e-system group were aggravating factor (27/365, 7.4%), aggravation by increased intracranial pressure (70/365, 19.2%), and relieving factors (110/365, 30.1%), while onset (311/365, 85.2%), vomiting (298/365, 81.6%), and duration (287/365, 78.6%) were the features with the highest level of data acquisition. All clinical features were obtained in over 90% (range 410/452, 90.7% to 452/452, 100%) of patients in the after e-system group. Timing (410/452, 90.7%), location (446/452, 98.7%), and severity (450/452, 99.6%) were the 3 least obtained clinical features after the implementation of the e-system (Table 1).

**Table 1.** Comparison of data acquisition rate between before and after introduction of the electronic questionnaire system (BEST-Survey^a^).

Clinical features	Before e-system (n=365), n (%)	After e-system (n=452), n (%)
Severity (NRS^b^ score)	196 (53.7)	450 (99.6)
Onset	311 (85.2)	452 (100)
Frequency	285 (78.1)	452 (100)
Duration	287 (78.6)	451 (99.8)
Location	248 (67.9)	446 (98.7)
Characteristics	221 (60.5)	452 (100)
Nausea	276 (75.6)	452 (100)
Vomiting	298 (81.6)	452 (100)
Dizziness	263 (72.1)	452 (100)
Photophobia	174 (47.7)	452 (100)
Phonophobia	156 (42.7)	452 (100)
Visual aura	130 (35.6)	452 (100)
Timing	228 (50.9)	410 (90.7)
Sleep breakage	219 (60.0)	452 (100)
Relieving factors	110 (30.1)	452 (100)
Aggravating factors	27 (7.4)	452 (100)
Aggravation by IICP^c^	70 (19.2)	452 (100)
Family history	133 (36.4)	452 (100)

a BEST-Survey: Bundang Hospital Electronic System for Total Care-Survey.

b NRS: Numeral Rating Scale.

c IICP: increased intracranial pressure.

### Clinical Characteristics of Headaches From Patients Who Visited Our Hospital After the Implementation of BEST-Survey

Detailed clinical headache characteristics of our pediatric patient group was acquired from the BEST-Survey system. In our patient group, most patients (280/452, 61.9%) had their onset of headache less than or equal to 1 year ago, and they most frequently complained of daily headaches (116/452, 25.6%). The temporal region was the most common location of headaches (261/703, 37.1%; unilateral: 111/703, 15.8% and bilateral 150/703, 21.3%), followed by the frontal head region (111/703, 15.8%). Pain characteristics were often pulsatile (188/861, 21.8%), pressing (140/861, 16.3%), or dull (159/861, 18.5%). More than half of patients (232/452, 51.3%) had headaches lasting less than 2 hours, with nausea and vomiting (231/1036, 22.3%) as the most commonly associated symptoms. Red-flag symptoms were reported by 316 (69.9%) out of 452 patients. Of the patients reporting red-flag symptoms, sleep breakage and improvement by vomiting accounted for 137 (26.5%) and 71 (13.7%) out of 517 red-flag symptoms, respectively. The clinical characteristics of the patients with headaches obtained from BEST-Survey are shown in Table 2.

**Table 2.** Clinical characteristics of headaches obtained from BEST-Survey^a^. Multiple answers were allowed in some questions.

Clinical characteristics	Value, n (%)
**Headache onset (n=452 patients)**	
Within 1 month	70 (15.5)
Within 3 months	77 (17)
Within 6 months	50 (11.1)
Within 1 year	83 (18.4)
Within 2 years	70 (15.5)
Within 3 years	44 (9.7)
Within 4 years	21 (4.6)
Within 4 years	13 (2.9)
>5 years	24 (5.3)
**Headache frequency (n=452 patients)**	
Daily	116 (25.6)
3‐4 times/week	74 (16.3)
1‐2 times/week	89 (19.6)
1‐3 times/month	76 (16.8)
<1 time/month	29 (6.4)
Not definite	68 (15)
**Headache nature (n=452 patients; multiple answers allowed: n=861 answers)**	
Stabbing	26 (3)
Pressing	140 (16.3)
Tightening	151 (17.5)
Pulsatile	188 (21.8)
Aching	99 (11.5)
Dull	159 (18.5)
Not definite	98 (11.4)
**Headache duration (n=452 patients)**	
<30 minutes	76 (16.8)
30 minutes to 1 hour	89 (19.7)
1-2 hours	67 (14.8)
2–3 hours	52 (11.5)
3–4 hours	39 (8.6)
4–5 hours	11 (2.4)
5–6 hours	14 (3.1)
6–12 hours	24 (5.3)
12–24 hours	23 (5.1)
>24 hours	12 (2.7)
Unspecified	45 (10)
**Headache location (n=452 patients; multiple answers allowed: n=703 answers)**	
Whole	80 (11.4)
Bilateral temporal	150 (21.3)
Unilateral temporal	111 (15.8)
Vertex	74 (10.5)
Occipital	90 (12.8)
Frontal	111 (15.8)
Around eyes	59 (8.4)
Neck	21 (3)
Not definite	7 (1)
**Associated symptoms (n=452 patients; multiple answers allowed: n=1036 answers)**	
Nausea or vomiting	231 (22.3)
Irritability	183 (17.7)
Distraction	177 (17.1)
Transient amnesia	6 (0.6)
Sensitivity to lights or sounds	213 (20.6)
Cramping	25 (2.4)
Tearing or ptosis	56 (5.4)
Yawning	72 (6.9)
Increased urination	12 (1.2)
Diarrhea	14 (1.4)
Depression	47 (4.5)
**Aura (n=452 patients; multiple answers allowed: n=405 answers)**	
Vertigo	14 (3.5)
Dizziness	84 (20.7)
Ataxia	15 (3.7)
Altered consciousness	5 (1.2)
Motor aura	38 (9.4)
Language aura	9 (2.2)
Sensory aura	4 (1)
Auditory aura	105 (25.9)
Visual aura	113 (27.9)
Not definite	18 (4.4)
**Red-flag symptoms (n=316 patients; multiple answers allowed: n=517 answers)**	
Headache after hyperventilation	154 (29.8)
Sleep breakage	137 (26.5)
Improved by vomiting	71 (13.7)
Morning headache	155 (30)

a BEST-Survey: Bundang Hospital Electronic System for Total Care-Survey.

## Discussion

### Principal Findings in Comparison to Prior Works

In this study, we developed an electronic questionnaire system named BEST-Survey and utilized it to collect medical history from patients with pediatric headaches. This system, which includes a streamlined previsit electronic headache questionnaire, automatic integration into the EHR, and construction of a structured database for further analysis, was significantly effective in ensuring the completeness of collecting the key clinical features of pediatric headaches. Furthermore, the system allowed easy data extraction and analysis for the comprehensive clinical characterization of pediatric headaches. To the best of our knowledge, this is the first study describing an integrated electronic questionnaire system linked to an EHR for pediatric headache.

Traditional history taking involves interactive conversations with patients to establish rapport, assess communication skills, observe their condition, and collect relevant clinical information [22]. However, comprehensive history taking could be hindered by miscommunication between a patient or parent and their physician, as well as the limited time available. Inquiring all necessary questions and retrieving accurate answers is crucial for accurate diagnosis and appropriate management [23]. Omitting important questions can significantly impact diagnosis and treatment outcomes [24]. Moreover, the time-consuming EHR documentation process further hinders accurate history taking and medical care delivery.

Studies on ambulatory practices have shown that physicians spend 18% to 20% of their time on documentation or writing [25]. Another study revealed that 52.9% of their time is dedicated to direct clinical face time, while 37% is spent on EHR and desk work [26]. Taking medical histories from children poses unique challenges compared to adults. Limited expression of symptoms and reliance on parents or guardians for information [27], as well as the potential for distraction and poor cooperation from the children [28], require detailed explanations and additional time for history taking. To address the limitations to accurate history taking, our system offers several advantages. First, a predetermined questionnaire form ensures that essential data are gathered without omitting important questions. Second, utilizing the waiting time before a consultation gives the patients more time to report their symptoms. Third, automatic integration with the EHR saves efforts required for documentation. Additionally, the computerized and stored data facilitate further processing and analysis.

### Strengths of the Streamlined Electronic Questionnaire System

Our system’s advantage in capturing essential history items without omission was demonstrated through a comparison of data acquisition rates before and after its implementation. The findings revealed a significant increase in obtaining answers to each item after introducing our system. Prior to using the system, interviews alone resulted in less than 90% of patients providing essential headache diagnosis information such as onset time, frequency, duration, location, and characteristics. Furthermore, inquiries regarding factors worsening the condition, increased intracranial pressure, and relief had the lowest response rates. These results align with previous studies demonstrating the efficacy of digital questionnaires in enhancing data collection. In a systematic review examining the benefits of electronic patient-reported outcome measures, 10 (31%) out of 32 studies reported having missing or incomplete data. Among those 10 studies, 7 (70%) reported lower rates of missing data and more complete information with electronic methods compared to paper-based approaches [19]. Another study comparing histories acquired by physicians and a computer program directly interacting with patients found that the computer-acquired histories revealed 160 problems not documented by physicians. Conversely, there were 13 problems reported by physicians but not by the computer program, indicating the usefulness of computer programs in acquiring more comprehensive and detailed medical histories [29].

We also examined differences in the rate of positive findings for each history item. We observed variations in the rate of positive responses, with interview-based inquiries generally yielding higher positive rates, except for sleep breakage. The high positive rates of various clinical characteristics obtained through physician interviews may be due to discrepancies and inaccuracies in reflecting the actual phenomenon. For instance, in our study, the proportion of patients with photophobia was 41.4% in the before e-system group and 12.2% in the after e-system group. In previous studies of patients with pediatric headaches in South Korea, 43% were diagnosed with migraines, 35% had tension headaches, and 22% had other primary headaches [30]. Considering that approximately half of patients with migraines experience photophobia [31], the finding of 41.4% of patients with photophobia in our study is likely an overestimation even when considering the higher likelihood of patients with debilitating headaches seeking care at tertiary hospitals. Discrepancies between physician interview–based information and patient-reported data have been reported in previous studies. In an analysis of clinical interviews and computer-acquired history data among patients with chest pain, inconsistencies were observed in the collected data [32]. Both our study and previous studies on chest pain have consistently found higher levels of missing data when obtaining medical history through interviews compared to using e-questionnaires or computerized history taking. The primary reason for these discrepancies can be attributed to data incompleteness during the interview-based, history-taking process.

Another advantage of the proposed system in this study is that the computerization of data enables easy data processing, analysis, and storage for future use. This facilitates efficient data analysis and saves processing time. For instance, we conducted an analysis on the clinical characteristics of patients with headaches visiting our hospital, providing an overview of their features. In our study population, predominant headache characteristics included daily occurrence (25.7%), severity rating around an NRS score of 7‐8 (40.9%), pulsatile nature (21.6%), onset between 6 months to 1 year (18.4%), duration less than 4 hours (71.5%), and involvement of the entire or bilateral temporal area of the head (37.1%). Several studies have evaluated headache characteristics in children attending headache outpatient clinics. In one study with a questionnaire collected from 437 pediatric patients referred to headache outpatient clinics, 5.9% had underlying organic diseases, while 94% had primary headaches [33]. The characteristics of patients with primary headaches analyzed in this study showed similarities to our study, including a significant proportion of patients with headache duration between 2‐12 hours (68.8%), a high incidence of bilateral pain (63.9%), and a high frequency of severe intensity ratings (63.9%) and pulsatile features (27.1%). The incidence of accompanying symptoms such as nausea (49.1%), vomiting (37%), photophobia (27.8%), phonophobia (24.4%), and aura (13.5%) was similar or slightly higher compared to our study population. Another study of 194 pediatric patients with primary headaches presenting to an outpatient clinic in Jordan also showed similarities to the headache characteristics in our study. The main patient population experienced moderate to severe headaches (80.4%), daily occurrence (34.5%), duration of 0.5‐4 hours (25.5%), and a higher proportion of bilateral pain (78.9%). The prevalence of accompanying symptoms, including nausea or vomiting (43.1%), dizziness (31%), and photophobia (38.8%), was similar or slightly higher than those in our study [34]. In this study, we included both primary and secondary headaches, whereas previous studies solely focused on primary headaches. However, due to the relatively small proportion of patients with secondary headaches within the overall group of patients with headache patients, similar results were obtained.

We were also able to identify key considerations for implementing the EHR-integrated, e-questionnaire system in clinical practice. First, using plain language in the questions is crucial as they are asked without additional explanations. However, care must be taken to avoid excessive simplification, which can result in a higher false positive rate. Therefore, continuous updating of the questions is necessary to minimize discrepancies between physician-led history taking and patient-reported questionnaires. For example, the question “Is there weakness in the arms or legs?” is intended to assess symptoms of transient ischemic attacks, a key symptom of Moyamoya disease. However, patients often misinterpret this question as fatigue or general weakness. Thus, more detailed questions differentiating between fatigue and paralysis are necessary. Second, the system should serve as a supplement to, rather than a replacement for, physician-led history taking. Double-checking items marked positively by the patient through the questionnaire is necessary. Third, appropriate question styles should be considered for different age groups to compensate for different levels of understanding. Tailored questionnaires for different age groups are essential for the success of electronic questionnaire system in the clinical field.

### Limitations and Future Directions

Our study has several limitations. First, we did not directly compare the history-taking methods used by physicians with the electronic system for the same patient simultaneously. Second, we did not assess the validity of the questionnaire. Third, we did not evaluate whether the introduction of the system resulted in clinically significant changes in diagnosis and management. Based on our limitations, we suggest the following future studies utilizing the system developed in this study. First, a study should be conducted to assess the system’s validity by comparing data obtained from physician-led history taking and patient-completed questionnaires for the same individuals. Second, investigations are needed to examine the impact of the system on patient care and outcomes in real-world clinical settings. Third, the development of a clinical decision support system based on the electronic system should be explored. Fourth, the development of our survey was conducted internally and did not undergo validation by external reviewers. Due to this lack of validation, our survey maybe limited in detailed characterization for certain subtypes of headaches, such as tension-type headaches. Fifth, the lack of investigation into the time spent to complete the questionnaire limits us in comparing the total saved clinic time. Sixth, the lack of quantitative feedback from patients, families, and clinicians about the acceptability of the electronic questionnaire limits us in understanding the acceptability of the system. Finally, future studies could investigate the improved diagnostic rate of headache subtypes following system implementation and explore changes in headache burden after administering various medications. A streamlined questionnaire could enhance the completeness of phenotypic data, facilitating the diagnosis of complex, rare diseases and potentially shortening their diagnostic odyssey. Our proposed system could also be adapted for use in other disease contexts [35]. Such studies would help demonstrate the qualitative improvement of the information collected by our electronic questionnaire system.

### Conclusion

In this study, we developed an electronic questionnaire system named BEST-Survey and utilized it to implement a patient-reported electronic questionnaire for patients with pediatric headaches. A streamlined, previsit electronic questionnaire that is automatically integrated to the EHR showed several strengths over traditional interview-based history taking. First, it ensures the completeness of essential history items. Second, it enables more accurate history taking, particularly in key clinical features that may be overestimated in traditional methods. Finally, the system facilitates easy data extraction, processing, and analysis, enabling detailed clinical characterization of the specific patient population of interest. While the electronic questionnaire system cannot replace the complex role of physician-led history taking, it can serves as a helpful adjunctive tool to improve patient care.

## Supplementary material

10.2196/54415 Multimedia Appendix 1 List of questions (in English and Korean).

## Abbreviation

BEST-Survey: Bundang Hospital Electronic System for Total Care-Survey
EHR: electronic health record
IRB: institutional review board
NRS: Numeric Pain Rating
