# Supplementary material for: Enhancing Clinical History Taking Through the Implementation of a Streamlined Electronic Questionnaire System at a Pediatric Headache Clinic: Development and Evaluation Study
Source: JMIR Med Inform. 2024 Nov 8;12:e54415. doi: 10.2196/54415 (PMC11611800; doi:10.2196/54415)
Supplement: Multimedia Appendix 1 [file medinform-v12-e54415-s001.docx]

**Multimedia Appendix 1**

**Table S1. List of Questions (English)**

| **Question Category** | **Question and Answer** |
| --- | --- |
| **Remote onset** | **Q1. When did the headache start?** |
|  | □ <1 month □ <3 months □ <6 months □ < 1 year □ <2 years □ <3 years □ <4 years □ <5 years  □ If more than 5 years or if you know the exact date, please specify |
| **Recent aggravation** | **Q2. Has your headache worsened recently?** |
|  | □ Yes □ No □ I don’t know |
| **Recent aggravation since** | **Q3. When did it get worse?** |
|  | □ <1 week □ <2 weeks □ <4 weeks □ <3 months □ <6 months □ If not applicable, please specify |
| **Aggravation of** | **Q4. Please select all of the aspects of your headache that have worsened.** |
|  | □ Frequency □ Intensity □ Duration □ If not applicable, please specify ( ) |
| **Specific situation at onset** | **Q5. Did any of conditions below preceded to your headache? Please select all of them.** |
|  | □ Head trauma □ Accidents other than head trauma □ Excessive stress □ Fever □ If not applicable, please specify ( ) |
| **Headache pattern** | **Q6. How has your headache been recently (one month or months)? Please select all that apply.** |
|  | □ Intermittent, isolated headaches that does not get worse (completely pain-free when there is no headache)  □ Intermittent, isolated headaches that gradually worsens in intensity, frequency, or duration (completely pain-free when there is no headache)  □ Intermittent daily headache  □ Daily persistent headache  □ If not applicable, please specify ( ) |
|  | **Q7. Please describe the type of headache in as much detail as possible. Please select all that apply**. |
|  | □ Stabbing □ Pressing □ Tightening □ Pulsating □ Throbbing □ Aching □ Dull □ If not applicable, please specify ( ) |
| **Severity, NRS** | **Q8. Express the intensity of your headache as a number from 0 to 10.** |
|  | □ 0 □ 1 □ 2 □ 3 □ 4 □ 5 □ 6 □ 7 □ 8 □ 9 □ 10 |
|  | **Q9. For infants who cannot express their pain in NRS, how do you as a parent rate the pain when your child has headache?** |
|  | □ It doesn't seem very painful □ looks a little sick □ looks very sick □ If not applicable, please specify ( ) |
| **Location** | **Q10. When you have a headache, where does it hurt the most? If you point with your finger, where is it? Please select all that apply.** |
|  | □ Whole head □ Side heads (both) □ Left side head □ Right side head □ Top of the head □ Back of the head □ Neck □ Forehead □ Around eyes □ If not applicable, please specify ( ) |
| **Duration** | **Q11. How long do headaches last on average until the complete resolution?** |
|  | □ <30 minutes □ 30minutes-<1 hour □ 1-2 hours □ 2-3 hours □ 3-4 hours □ 4-5 hours □ 5-6 hours □ 6-12 hours □ 12-24 hours □ ≥24 hours □ If not applicable, please specify ( ) |
| **Frequency** | **Q12. How often do headaches occur on average?** |
|  | □ More than once a day □ 3-4 times per week □ 1-2 times per week □ 1-3 times a month □ Less than once a month □ If not applicable, please specify ( ) |
| **HA occurs frequently at** | **Q13. Are there times when headaches occur often? Please select all that apply.** |
|  | □ Waking up □ morning □ around lunch □ afternoon □ evening □ before sleep □ none  □ If not applicable, please specify ( ) |
| **Aggravating factor** | **Q14. Which of the following worsens your headaches? Please select all that apply.** |
|  | □ Specific food □ Going to school □ Running □ Climbing stairs □ Car ride □ Coughing □ Straining on the toilet □ Severe stress □ If not applicable, please specify ( ) |
| **Alleviating factors** | **Q15. Which of the following relieves your headaches? Please select all that apply.** |
|  | □ Sleep □ Rest □ Lying down □ Standing □ Dark place □ Quite place □ If not applicable, please specify ( ) |
| **Aura** | **Q16. Do you have an aura before your headache?** |
|  | □ Yes □ No |
| **Specific aura** | **Q17. If you have an aura, please select all that apply to you below. If necessary, please describe in more detail.** |
|  | □ Black dots are seen □ Object looks distorted □ Flashing □ Blurring □ One object seen as two □ Sensory change □ Can't remember the word □ Slurred speech □ Tinnitus □ Hearing difficulty (difficulty hearing) □ Shaking gait □ Altered consciousness □ Dizziness □ Vertigo (whirling vision) □ Weakness of arms or legs □ If not applicable, please specify ( ) |
| **Associated symptom** | **Q18. If you have symptoms that accompany a headache, please select all that apply from the following.** |
|  | □ Nausea □ Vomiting □ Irritability □ Attention deficit (difficulty concentrating) □ Transient memory loss □ Difficult to find a way □ Photophobia (the light makes the headache worse) □ Phonophobia (  headache aggravated by loud noises) □ Osmophobia (sensitive to smell) □ Hyperalgesia (feeling pain even with minor touch) □ Muscle pain □ Tearing □ Ptosis □ Yawn □ Frequent urination (want to go to bathroom often) □ Diarrhea □ Depressive mood |
| **Sleeping tendency** | **Q19. Are you sleepy when you have a headache?** |
|  | □ Yes □ No |
| **Improved by sleep** | **Q20. Does the headache go away after sleeping?** |
|  | □ Yes □ No |
| **Worsening by lying down** | **Q21. When your head hurts, does your head hurt more when you lie down?** |
|  | □ Yes □ No |
| **Worsening by standing** | **Q22. When your head hurts, does your head hurt more when you stand up?** |
|  | □ Yes □ No |
| **Morning severe headache** | **Q23. Did you ever had a severe headache when you woke up in the morning?** |
|  | □ Yes □ No |
| **Improved by vomiting** | **Q24. If it is accompanied by vomiting, does the headache improve after vomiting?** |
|  | □ Yes □ No |
| **Worsening by Valsalva** | **Q25. Does leaning forward or straining your stomach make your headache worse?** |
|  | □ Yes □ No |
| **Sleep breakage** | **Q26. Have you ever woken up with a headache?** |
|  | □ Yes □ No |
| **Headache after hyperventilation** | **Q27. Do you get headaches from hyperventilation, such as when you exercise or blow on hot food or blow a musical instrument?** |
|  | □ Yes □ No |
| **Underlying disease** | **Q28. If any of the following diseases have been diagnosed, please select all that apply.** |
|  | □ Cancer, leukemia □ Hemorrhagic disease □ Rheumatic disease □ Congenital heart disease □ Hypertension □ If not applicable, please specify ( ) |
| **Family history of headache** | **Q29. Has anyone in your family been diagnosed with headaches?** |
|  | □ Yes □ No |
| **Family member with headache** | **Q30. If you have a family history, please select all that apply to you.** |
|  | □ Paternal grandfather □ Paternal grandmother □ Maternal grandfather □ Maternal grandmother □ Father □ Mother □ Brother □ Sister □ If not applicable, please specify ( ) |
| **Effects on daily lives** | **Q31. Does headaches affect your daily life? (eg, going to school, eating, kindergarten/school activities, playing, etc.)** |
|  | □ No □ Mild □ Moderate □ Severe □ If not applicable, please specify ( ) |
| **Previous work-up** | **Q32. Have you been evaluated for headaches before?** |
|  | □ Yes □ No |
| **Previous work-up type** | **Q33. If you have had any tests, please select all of the following tests.** |
|  | □ Brain CT □ Brain MRI □ EEG □ Cerebral arterial blood flow test □ If not applicable, please specify ( ) |
| **Previous treatment** | **Q34. Have you ever been treated for a headache before?** |
|  | □ Yes □ No |
| **Further history and patient’s remarks** | **Q35. If you have any additional comments, special concerns, or questions, please write them down.** |

**Table S2. List of Questions (Korean)**

| **Question Category** | **Question and Answer** |
| --- | --- |
| **Remote onset** | **Q1. 두통이 언제부터 시작되었나요?** |
|  | 1개월 이내, 3개월 이내, 6개월 이내, 1년 이내, 2년 이내, 3년 이내, 4년 이내, 5년 이내, 기타(5년 이상이거나 정확한 시기를 아는 경우 기입해주세요) |
| **Recent aggravation** | **Q2. 최근 두통이 악화되었나요?** |
|  | 예, 아니오, 잘 모르겠음 |
| **Recent aggravation since** | **Q3. 악화되었다면 언제부터인가요?** |
|  | 1주 이내, 2주 이내, 4주 이내, 3개월 이내, 6개월 이내, 기타 |
| **Aggravation of** | **Q4. 두통의 어떤 점이 악화되었는지 모두 선택해 주세요.** |
|  | 빈도, 강도, 지속 시간, 기타 |
| **Specific situation at onset** | **Q5. 두통이 처음 발병하였을 때 다음에 해당하는 특별한 일이 있었다면 모두 선택해 주세요.** |
|  | 두부 외상(머리 다침), 두부 외상 외 다른 사고, 과도한 스트레스, 발열, 기타 |
| **Headache pattern** | **Q6. 최근(1개월 혹은 수개월 간) 두통의 양상은 어떤가요? 해당되는 것을 모두 선택해 주세요.** |
|  | 간헐적으로 한 번 씩 아프고 악화되지 않음(두통이 없을 때는 증상이 완전히 없음), 간헐적으로 한 번 씩 아픈데 점점 강도, 빈도, 또는 지속 시간이 악화됨(두통이 없을 때는 증상이 완전히 없음), 매일 두통이 발생하고 하루를 볼 때 간헐적으로 아픔, 매일, 하루 종일 아픔 (한 시도 안 아픈 적이 없음), 기타 |
|  | **Q7. 두통의 양상을 최대한 자세히 설명해주세요. 해당되는 것을 모두 선택해 주세요.**  **표현이 자세하지 않은 소아의 경우 환자가 표현하는 대로 적어주세요.** |
|  | 칼로 찌르는 듯이 날카롭다, 무겁게 누른다, 띠를 두른 듯 조인다, 심장 뛰듯이 박동성으로 아프다, 머리가 울린다, 맞은 듯이 아프다, 머리가 무겁다(그냥 불편하다), 기타 |
| **Severity, NRS** | **Q8. 두통의 강도를 숫자로 표현한다면, 가장 아플 때 몇 점까지 아픕니다. 10점은 본인이 경험한 통증 중 가장 심한 통증이고 전혀 아프지 않은 것이 0점입니다.** |
|  | □ 0 □ 1 □ 2 □ 3 □ 4 □ 5 □ 6 □ 7 □ 8 □ 9 □ 10 |
|  | **Q9. 점수로 강도를 알기 어려운 소아의 경우 부모님이 보시기에 어떤지 아래에서 골라 주시기 바랍니다.** |
|  | 별로 아프지 않아 보인다, 조금 아파 보인다,많이 아파 보인다, 기타 |
| **Location** | **Q10. 두통이 있을 때 가장 아픈 위치는 어디입니까? 손가락으로 가리킨다면 어디인지요? 해당되는 것을 모두 선택해 주세요.** |
|  | 머리 전체, 옆 머리(양쪽), 왼쪽 옆머리, 오른쪽 옆머리, 정수리(머리 꼭대기), 뒷머리, 목덜미, 이마, 눈 주변, 기타 |
| **Duration** | **Q11. 두통은 평균적으로 얼마나 오랫동안 지속되나요? 한 번 발생하면 완전히 소실되기까지 걸리는 시간 입니다.** |
|  | 30분 이내, 30분~1시간, 1시간~2시간, 2시간~3시간, 3시간~4시간, 4시간~5시간, 5시간~6시간, 6시간~12시간, 12시간~24시간, 24시간 이상, 기타 |
| **Frequency** | **Q12. 두통은 평균적으로 얼마나 자주 발생합니까?** |
|  | 하루 1회 이상, 주당 3~4회, 주당 1~2회, 한달에 1~3회, 한달에 1회 미만, 기타 (더 정확한 횟수를 알거나 위 보기로 설명하기 어려운 경우 기입해주세요) |
| **HA occurs frequently at** | **Q13. 두통이 주로 발생하는 시간대가 있습니까?해당되는 것을 모두 선택해 주세요.** |
|  | 자고 일어나서, 오전, 점심 즈음, 오후, 저녁 즈음, 자기 전, 없다, 기타 |
| **Aggravating factor** | **Q14. 다음 중 두통을 더 자주 유발시키는 요인이 있나요?해당되는 것을 모두 선택해 주세요.** |
|  | 특정 음식, 학교 등교, 달리기, 계단 오르기, 등산, 오래 서 있기, 자동차 타기, 기침, 용변 보기, 심한 스트레스, 기타 |
| **Alleviating factors** | **Q15. 다음 중 두통을 완화시키는 요인이 있나요?해당되는 것을 모두 선택해 주세요.** |
|  | 수면, 휴식, 누운 자세, 서있는 자세, 어두운 곳, 조용한 환경, 기타 |
| **Aura** | **Q16. 머리가 아프기 전에 보이는 것이 이상하거나 느낌이 이상하거나 하는 전조 증상이 있어 본인이 알 수 있는지요?** |
|  | 예, 아니오 |
| **Specific aura** | **Q17. 전조 증상이 있다면 다음에서 해당되는 것을 모두 선택해 주세요. 필요한 경우 기타에 자세히 기술해 주세요.** |
|  | 까만 점이 보임, 물체가 일그러져 보임, 밝은 빛(번쩍거림)이 보임, 눈이 안 보임, 물체가 뿌옇게 보임, 물체가 두 개로 보임, 감각 변화, 단어가 생각이 나지 않음, 말이 어눌해짐, 이명(구에서 소리가 남), 청력 장애(소리가 잘 안 들림), 비틀거림, 의식 저하, 어지러움, 현훈(자신이나 주변이 빙글빙글 돔), 팔이나 다리의 힘 빠짐, 기타 |
| **Associated symptom** | **Q18. 두통이 있을 때 동반되는 증상이 있으면 다음에서 해당되는 것을 모두 선택해 주세요.** |
|  | 구역감, 구토, 짜증내거나 보챔, 주의력 결핍(집중이 어려움), 일시적인 기억 손실, 길 찾기가 어려움, 광과민증(빛을 보면 두통이 악화됨), 소음 과민증(시끄러운 소리에 두통이 악화됨), 냄새 과민증, 통각 과민증(사소한 자극에도 통증을 느낌), 근육통, 눈물, 눈꺼풀 처짐, 하품, 잦은 요의(화장실 가고 싶음), 설사, 우울감 |
| **Sleeping tendency** | **Q19. 두통이 있을 때 졸립니까?** |
|  | 예, 아니오 |
| **Improved by sleep** | **Q20. 자고 나면 두통이 사라지나요?** |
|  | 예, 아니오 |
| **Worsening by lying down** | **Q21. 머리가 아플 때, 누우면 머리가 더 아픈가요?** |
|  | 예, 아니오 |
| **Worsening by standing** | **Q22. 머리가 아플 때, 일어서면 머리가 더 아픈가요?** |
|  | 예, 아니오 |
| **Morning severe headache** | **Q23. 아침 일찍 일어나자 마자, 극심한 두통이 있어서 학교를 못 가거나, 울거나 한 적이 있나요?** |
|  | 예, 아니오 |
| **Improved by vomiting** | **Q24. 구토가 동반되는 경우, 토하고 나면 두통이 호전되나요?** |
|  | 예, 아니오 |
| **Worsening by Valsalva** | **Q25. 머리가 아플 때, 앞으로 기울이거나 배에 힘을 주면 머리가 더 아픈가요?** |
|  | 예, 아니오 |
| **Sleep breakage** | **Q26. 머리가 아파서 자다가 깬 적이 있나요?** |
|  | 예, 아니오 |
| **Headache after hyperventilation** | **Q27. 심한 운동을 하거나 뜨거운 음식을 불었을 때, 혹은 악기를 불었을 때와 같이 과호흡을 하는 상황에서 두통이 발생하나요?** |
|  | 예, 아니오 |
| **Underlying disease** | **Q28. 다음 보기 중 진단 받은 질병이 있다면 해당되는 것을 모두 선택해 주세요.** |
|  | 암, 백혈병, 출혈성 질환, 류마티스 질환, 선천성 심장 질환, 고혈압, 기타 (그 외 진단받고 치료받고 있는 질환) |
| **Family history of headache** | **Q29. 가족을 포함한 직계 가족 중 두통으로 진단받은 사람이 있습니까?** |
|  | 예, 아니오 |
| **Family member with headache** | **Q30. 가족력이 있다면 해당되는 가족을 모두 선택해 주세요.** |
|  | 조부, 조모, 외조부, 외조모, 아버지, 어머니, 형제, 자매, 기타 |
| **Effects on daily lives** | **Q31. 두통이 일상 생활에 영향을 줍니까? (예, 등교, 식사, 유치원 활동, 놀이 등 )** |
|  | 전혀 영향을 주지 않는다., 영향을 준 적이 있다, 그러나 절반 이하 혹은 경미한 정도, 영향을 꽤 준다. 중간 이상, 심각한 지장을 초래한다., 기타 |
| **Previous work-up** | **32. 이전에 두통에 대해 검사를 받으신 적이 있습니까?** |
|  | 예, 아니오 |
| **Previous work-up type** | **Q33. 검사를 받으신 적이 있다면 다음 중 어느 검사를 받으셨는지 모두 선택해 주세요.** |
|  | 뇌 CT, 뇌 MRI, 뇌파, 뇌동맥혈류 검사, 기타 |
| **Previous treatment** | **Q34. 이전에 두통에 대해 치료를 받으신 적이 있습니까?** |
|  | 예, 아니오 |
| **Further history and patient’s remarks** | **Q35. 추가로 하시고 싶은 말씀, 특별히 걱정되시는 점, 궁금한 점이 있으시면 적어 주세요.** |
